# PCBs Make Their Mark: Review Pinpoints Cognitive Profile of Prenatal Exposure

**Published:** 2009-01

**Authors:** Carol Potera

Prenatal exposure to polychlorinated biphenyls (PCBs) impairs cognitive development in infants and children, according to numerous studies of these ubiquitous environmental pollutants. Studies of PCB damage have considered many different end points, but the results of these different studies have never been coordinated to pinpoint the neuropsychologic functions most likely to be damaged by prenatal exposure to PCBs. However, a review of longitudinal birth cohort studies in the medical literature reveals that impairment of executive functions—high-order brain processes responsible for planning, flexible thinking, abstract reasoning, problem solving, and inhibition of inappropriate actions—most consistently reflects prenatal PCB exposure **[*EHP* 117:7–16; Boucher et al.]**.

The review authors selected nine longitudinal birth cohort studies performed between 1959 to 2008 in North America, Europe, and Japan. Consumption of fish, whale blubber, and dairy products by pregnant women was the main source of prenatal PCB exposure as reflected by maternal serum concentrations that ranged from 23 to 450 ng/g of fat. All combined, about 4,000 children were monitored at different ages, from as early as 3 months to as late as 11 years, depending on the study. The types of tests conducted in the various studies included assessments of mental and psychomotor development of infants, IQ tests, and specific measures of verbal skills, visual–spatial ability, memory, attention, and executive functions. No one study measured all these neuropsychologic skills. Such a comprehensive evaluation would require a battery of complicated and expensive procedures.

The overall analysis found that executive functions are especially sensitive to PCB exposure. Three studies involving about 1,000 children specifically documented executive functions, and they all found that poor response inhibition was consistently related to prenatal PCB exposure. In one of these studies, children were exposed to some of the lowest doses of PCBs among the reviewed cohorts. Some of the studies reported that processes similar to executive functions—such as task planning, speed of information processing, verbal abilities, and visual recognition memory—were negatively impacted by prenatal exposure to PCBs as well. The authors conclude that executive functions in particular should be assessed in future cohort studies of the neurotoxic effects of PCBs and other organochlorine compounds.

